# Supplementary figures and images for: Gene Expression Profiling Elucidates Cellular Responses to NCX4040 in Human Ovarian Tumor Cells: Implications in the Mechanisms of Action of NCX4040
Source: Cancers (Basel). 2022 Dec 31;15(1):285. doi: 10.3390/cancers15010285 (PMC9818835; doi:10.3390/cancers15010285)

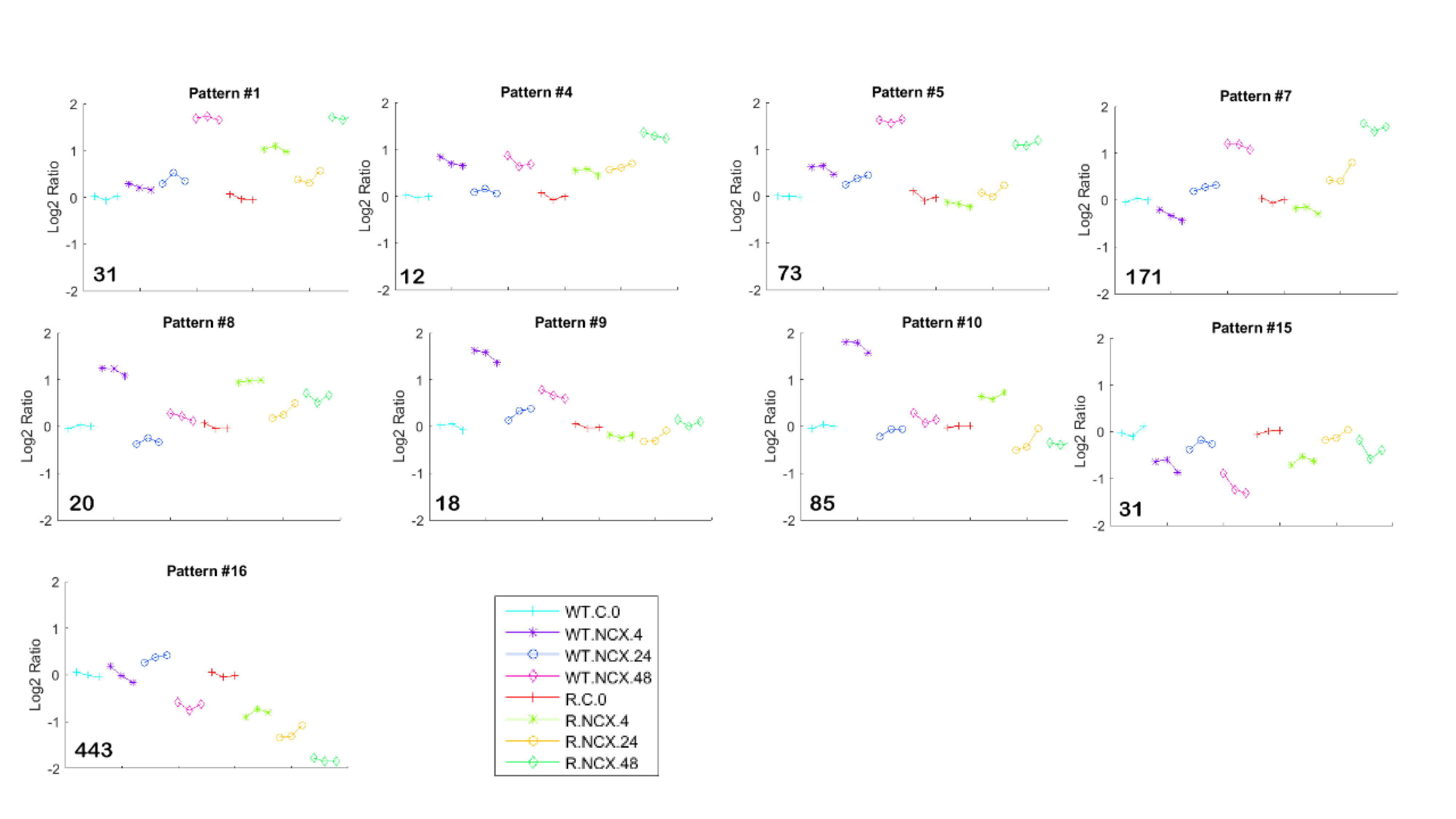

Supplement: Supplementary file 1 [file cancers-15-00285-s001.zip › Supplemental_Figure_S1_EPIG_9_patterns_not_discussed.jpg]
